# Retentive Strength of Orthodontic Bands Cemented with Amorphous Calcium Phosphate-Modified Glass Ionomer Cement: An In-Vitro Study

**Published:** 2017-01

**Authors:** Farzin Heravi, Maryam Omidkhoda, Niloufar Koohestanian, Tabassom Hooshmand, Hossein Bagheri, Negin Ghaffari

**Affiliations:** 1 Professor, Department of Orthodontics, School of Dentistry, Mashhad University of Medical Sciences, Mashhad, Iran; 2 Associate Professor, Oral & Maxillofacial Diseases Research Center, Mashhad University of Medical Sciences, Mashhad, Iran; 3 Advanced Implantology Student, UCLA School of Dentistry, Los Angles, USA; 4 Associate Professor, Department of Dental Biomaterials, School of Dentistry, Research Center for Science and Technology in Medicine, Tehran University of Medical Sciences, Tehran, Iran; 5 Assistant Professor, Dental Materials Research Center, Mashhad University of Medical Sciences, Mashhad, Iran; 6 Dental Student, University of Toronto, Faculty of Dentistry, Toronto, Canada

**Keywords:** Glass Ionomer Cements, Amorphous Calcium Phosphate, Retention

## Abstract

**Objectives:**

The aim of this study was to evaluate and compare the retentive strength of orthodontic bands cemented with amorphous calcium phosphate (ACP)-containing and conventional glass ionomer cements (GICs).

**Materials and Methods:**

One-hundred-and-twenty mandibular third molars were embedded in acrylic resin blocks with the buccal surface of crowns perpendicular to the base of the mold. The teeth were randomly divided into four groups containing 30 teeth each. Groups 1 and 3 were cemented using conventional GIC and groups 2 and 4 were cemented using ACP-containing orthodontic cement. Groups 1 and 2 without thermocycling, and groups 3 and 4 after thermocycling (5000 cycles, 5° to 55°C) were tested for retentive strength using a universal testing machine (crosshead speed of 1mm/minute). Two-way ANOVA was performed to compare the retentive strength of the groups.

**Results:**

The highest retentive strength belonged to group 1, and it was significantly higher than that of group 2 (P<0.001) and group 3 (P=0.02). The mean strength for group 2 was significantly lower than that of group 1 (P<0.001) and group 4 (P=0.04).

**Conclusions:**

Although retentive strength decreased when ACP was added to GIC, the retentive strength of the samples cemented by ACP-containing GIC was remarkably high after thermocycling. It seems that in the oral cavity, ACP-containing GIC provides sufficient strength to endure forces applied on posterior teeth.

## INTRODUCTION

Orthodontic bands are used as conventional orthodontic appliances placed around the crowns of posterior teeth to provide stable attachment for an arch wire [1]. Since the bands are placed in the posterior region, they are subjected to the greatest shear and tensile forces of mastication or trauma; therefore, their retention is crucial in order for an orthodontic treatment to be successful [2,3]. Optimally, the retentive strength of bands provided by the adhesive is sufficient to prevent debonding [3].

A significant issue associated with utilizing fixed appliances in orthodontics is demineralization of enamel adjacent to brackets and bands [4], which can be observed four weeks after the placement of fixed appliances [5]. The prevalence of demineralization in patients under orthodontic treatment by fixed appliances has been reported to be up to 96% [6]. Demineralization is seen more frequently at the margin of posterior bands; hence, presence of an orthodontic band, which enfolds a large area of tooth surface, makes tooth cleaning more difficult [7]. Therefore, orthodontic bands are believed to cause more enamel demineralization than brackets [8].

Different methods have been introduced to prevent or reduce enamel demineralization during orthodontic treatment [9]. In 1878, zinc phosphate cement was introduced as a dental cement [10] and over the years it has become the gold standard for orthodontic bands, and other orthodontic cements are compared to it [11,12]. Primary retention of zinc phosphate is due to mechanical bonds between the orthodontic band and tooth enamel. Moreover, it does not bond to enamel chemically [10]. High compressive strength, low tensile strength (making the cement brittle), short working time, and high solubility in the oral cavity (causing microleakage) are some of the drawbacks of zinc phosphate cement [13]. Fluoride was added to zinc phosphate cement in 1968 to reduce cement solubility and to impart anticariogenic properties to tooth enamel [14].

In 1968, zinc polycarboxylate was introduced as the first orthodontic cement that did not rely on mechanical retention to tooth enamel [11]. The ability to chemically bond to dental enamel and stainless steel suggests that polycarboxylate cements are suitable for use in orthodontic treatment [15]. Although, high viscosity, short setting and working times and high solubility make this cement less popular in orthodontic treatment [10,16].

Glass ionomer cements (GICs) are used as conventional adhesive materials in orthodontic treatment, which were first introduced in 1971 by Wilson and Kent [17]. Low solubility in the saliva, higher compressive and tensile strengths in comparison with zinc phosphate, acting as a chelator via an acid-base reaction in enamel and dentin to form a chemical bond to stainless steel, and the fluoride release potential are the favorable properties of GICs [13,18].

Amorphous calcium phosphate (ACP) can both be preventive and restorative [19,20]. These characteristics justify its incorporation in different dental products like toothpastes, prophylactic pastes, fluoride varnishes and gels, fissure sealants, anti-sensitivity agents, bleaching agents, dental cements, composites, and more recently, as an ingredient in orthodontic adhesives [12,21–24]. Also, ACP results in incorporation of nanocomplexes in the dental plaque and onto the tooth surface; thereby acting as a calcium and phosphate reservoir [25]. Rapid transfer of new minerals can remineralize the decalcified tooth surface. Regarding the ability of ACP to prevent demineralization, it seems that adding ACP to orthodontic cements could reduce white spot lesions (WSLs) during fixed orthodontic treatment, but the main concern is whether the use of ACP could alter mechanical properties of orthodontic cements.

The aim of this study was to assess the effect of incorporating ACP in GIC on its retentive strength when used for orthodontic band cementation. The null hypothesis tested was that there would be no difference in the mean retentive strength of orthodontic bands cemented with ACP-modified and conventional GICs.

## MATERIALS AND METHODS

In this experimental study, 120 extracted human mandibular third molars were used. The study protocol was approved in the ethics committee of Mashhad University of Medical Sciences (code: 89581). The criteria for tooth selection included intact buccal and lingual enamel with no cracks, no caries, no anomalies and no history of pretreatment of enamel surfaces with hydrogen peroxide, formalin, alcohol, or other chemical agents after extraction. The extracted teeth were cleaned, polished with non-fluoridated pumice paste and bristle brush, and then disinfected in 1% thymol for one week. All teeth were immersed in distilled water in a sealed container at room temperature until testing. The storage media were changed weekly in order to prevent the growth of microorganisms.

Standard stainless steel permanent mandibular first molar bands (Dentaurum, Pforzheim, Germany) with buccal tube and lingual sheath were selected and clinically adapted for best fit the crown of each tooth.

The roots of the teeth were embedded in a cylindrical mold using chemically cured acrylic resin. A surveyor was used to align the buccal surface of each tooth so that it was perpendicular to the base of the mold. The teeth were then cleaned with pumice slurry, washed in distilled water for 20 seconds, and dried in a stream of moisture-free air. The teeth were then randomly divided into four groups of 30 specimens each for band cementation.

The specimens were then cemented as follows:

Group 1: Bands were cemented to the teeth surfaces with GIC (Lot number 1109171; Gold label; GC Corporation, Tokyo, Japan). The cement was prepared according to the manufacturer’s instructions. It was loaded into the orthodontic band and each band was seated on each specimen with hand pressure using a stainless steel band seater. All the specimens were incubated in distilled water at 37°C for 48 hours.
Group 2: Bands were cemented to the teeth surfaces with ACP-containing GIC. Amorphous calcium phosphate was synthesized as follows: 46.3g of Ca(NO3)2, 4H2O was dissolved in 550mL of deionized water containing 40mL of 28 wt% ammonia. The solution was rapidly added to 27.2g of ammonium phosphate solution [(NH4)2HPO4 dissolved in 1300mL of deionized water containing 40mL of 28 wt% ammonia solution].
  Immediately after precipitation, the suspension was filtered and washed with deionized water containing 15mL of 28 wt.% ammonia solution and was finally lyophilized for 72 hours [26].
  Then, the ACP powder was kept in a freezer. Synthesized ACP powder was added to the GI powder (Lot number 1109171; Gold Label; GC Corporation, Tokyo, Japan) at 1.5% ratio [27]. All the specimens were incubated similar to group 1.
Group 3: All procedures were done similar to group 1. In addition, the specimens were subjected to thermocycling (5000 cycles in water baths between 5°C and 55°C with a dwell time of 30 seconds in each bath).
Group 4: All the procedures were done similar to group 2 and the specimens were subjected to thermocycling as described for group 3. Finally, all the specimens in the four groups were tested using a universal testing machine (Z250; Zwick Roell, Ulm, Germany) to assess the retentive strength at a crosshead speed of 1 mm/minute.

**Fig. 1:**
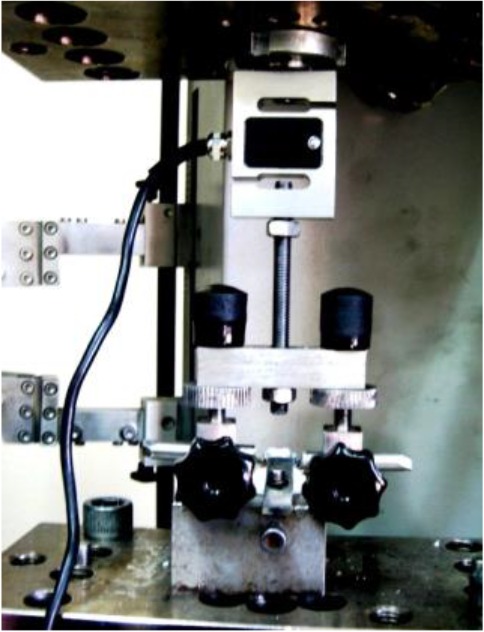
Mounted tooth clamped to a customized holding device fixed to the lower load cell of the universal testing machine

**Fig. 2:**
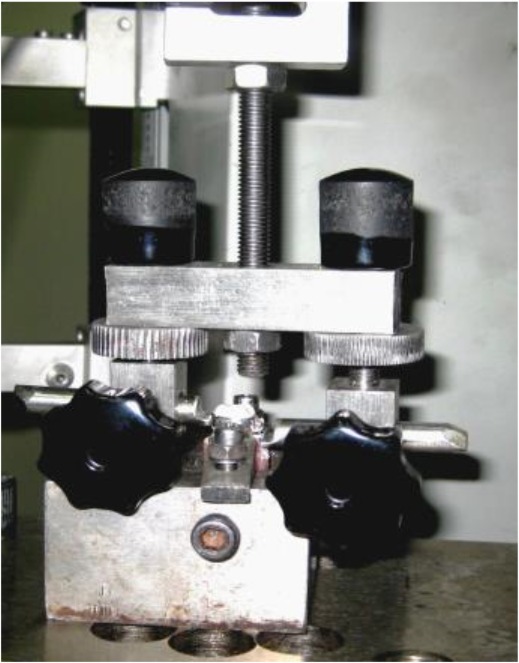
Customized band removal device used to simulate clinical load for measurement of retentive strength

Each tooth was mounted in a special jig and was clamped to a customized holding device, which was designed by the authors and fixed to the lower load cell of the universal testing machine (Fig. 1). The arrowheads of the holding device were engaged fully under the buccal tube and lingual sheath of each band (Fig. 2). This configuration allowed all forces to be directed parallel to the long axis of the tooth during debonding.

Testing proceeded for each specimen until the band was detached from the tooth. To calculate the bond strength values in megapascals (MPa), the maximum force recorded (in Newtons) during debonding was measured from the stress-strain curve for each specimen and then divided by the band surface area (in square millimeters; data provided by the manufacturer).

Descriptive statistics, including the mean, standard deviation, minimum and maximum values were computed for retentive strength for each group. Two-way ANOVA was used to determine significant differences between the groups.

## RESULTS

The Kolmogorov-Smirnov test was done to calculate the cumulative frequency (normalized by the sample size) before the statistical evaluation of retentive strength. All data were normally distributed. Table 1 shows the mean, standard deviation, minimum, maximum and range of retentive strength values in the test groups.

**Table 1:** Descriptive statistics (in megapascals) of the retentive strength (RS) of experimental groups (n=30)

**Material**	**Thermocycling**	**Mean**	**Standard deviation**	**Sig. (2-tailed)**
**GI**	Thermocycling	1.2961	0.35308	0.016
	No thermocycling	1.5140	0.32736	
**ACP**	Thermocycling	1.3691	0.25306	0.001
	No thermocycling	1.1695	0.18393	

GI: Glass ionomer; ACP: Amorphous calcium phosphate

Level of significance <0.05

**Fig. 3:**
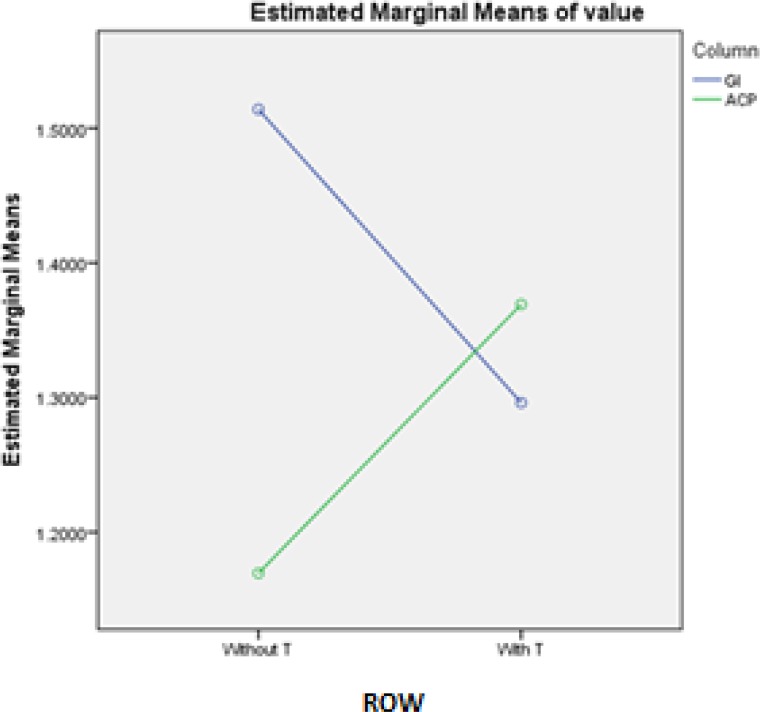
Interaction effect of type of glass ionomer and thermocycling (T) based on two-way ANOVA

Two-way ANOVA showed that the interaction effect of type of cement and thermocycling on retentive strength was significant (P<0.0001; Fig. 3). Independent t-test, as shown in Tables 2 and 3, demonstrated that thermocycling had no significant effect on retentive strength (P=0.36); although type of GIC without thermocycling had a significant effect on it (P=0.00).

**Table 2:** Independent samples t-test for evaluation of effect of thermocycling on retentive strength

**Group**	**Mean (MPa)**	**Std. Deviation**	**Std. Error**	**95% Confidence Interval for Mean**		**Minimum**	**Maximum**

				**Lower Bound**	**Upper Bound**		
Group 1	1.5140	0.32736	0.05977	1.3917	1.6362	0.92	2.40
Group 2	1.1695	0.18393	0.03358	1.1008	1.2382	0.70	1.57
Group 3	1.2961	0.35308	0.06446	1.1642	1.4279	0.52	2.09
Group 4	1.3691	0.25306	0.04620	1.2746	1.4636	0.97	1.94

**Table 3:** Independent samples test for comparison of retentive strength of groups

**Thermocycling**	**Material**	**Mean (MPa)**	**Standard Deviation**	**Sig.(2-tailed)**
**Thermocycling**	GI	1.2961	0.35308	0.36
	ACP	1.3691	0.25306	
**No thermocycling**	GI	1.5140	0.32736	0.00
	ACP	1.1695	0.18393	

Level of significance <0.05

GI: Glass ionomer; ACP: Amorphous calcium phosphate

The mean retentive strength in GIC groups decreased after thermocycling (P=0.016); on the other hand, the mean value of retentive strength increased in ACP modified-GIC group (P=0.001).

## DISCUSSION

The WSLs are undesirable but common complications of fixed orthodontic treatment. Their high prevalence, especially under posterior bands, obliges the orthodontists to use anticariogenic materials in conjunction with adhesives and cements. The development of ACP-containing materials such as casein phosphopeptide-amorphous calcium phosphate (CPP-ACP) in dentistry has drawn attention to this class of materials because of their numerous advantages [28–31]. Moreover, numerous studies have shown that ACP-containing materials have an important role in remineralization of WSLs and preventing enamel demineralization [9,23]. Recently, CPP-ACP has been added to different orthodontic adhesives and cements due to its anticariogenic and remineralization potential [25,31–34]. Although the rate of WLSs is high in posterior teeth and at the border of bands, studies on anticariogenic materials mostly focused on orthodontic brackets and their bond strength to the enamel surface [9,10].

Uysal et al, [9] indicated that ACP-containing orthodontic composite for bonding orthodontic brackets successfully inhibited enamel demineralization. Some studies have investigated the incorporation of ACP and CPP-ACP into orthodontic cements for banding, although the potential adverse effects of adding such materials to the cements remain unclear [9,10]. Therefore, in our study, we evaluated the retentive strength of GIC containing ACP for banding. Thirty third molar teeth were used per cement group. Orthodontic bands were cemented using conventional or ACP-containing GICs, and retentive strength of the bands was evaluated. The effect of thermocycling was also assessed.

In order to enhance the anticariogenic potential of GIC, we added 1.5% w/w ACP to a conventional GIC. Mazzaoui et al, [27] stated that incorporating 1.5% w/w CPP-ACP into a GIC yielded the best mechanical results. In our study, the highest mean retentive strength was found in group 1, which was cemented by GIC without thermocycling (1.514MPa), and it was comparable to the reported values of micro-etched bands cemented with GIC without thermocycling in studies by Millett et al, [1] (1.45MPa) and Wood et al, [35] (1.2 to 1.5MPa).

Unlike bracket bond strength [36], the normal range for retentive bond strength has not been stated in the literature. Consequently, our findings were compared to those of previous studies.

Uysal et al, [8] examined the shear bond strength of ACP-containing cement (Aegis Ortho) and found no statistically significant difference with that of GIC (Ketac-Cem). On the other hand, in our study the mean retentive strengths of bands cemented with the two cements differed significantly. The retentive strength of orthodontic bands cemented to the teeth with ACP-containing GIC was significantly lower than that of conventional GIC. One possible contributory factor is the different testing methods used in each study. In our study, tensile load was applied. The lowest amount of retentive bond strength was observed in group 2 (ACP-containing GIC without thermocycling), which could be caused by the fact that ACP particles are incapable of reinforcing composite structure, but based on a previous study, it is assumed that this amount of retentive strength is acceptable for clinical purposes [37]. In our study, the retentive strength of bands cemented with GIC significantly decreased after thermocycling (group 3) compared to group 1, which could be due to the effect of thermal stresses on the structure of GIC. This reduction was also reported in other studies [10,38,39]. Although adding ACP to GIC significantly decreased the retentive strength, an important finding was that after thermocycling, the retentive strength values in bands cemented with ACP-containing GIC (group 4) exceeded the values in bands cemented with conventional GIC (group 3). However, this difference was not significant. Similarly, in a study by Xiaojun et al, [40] the highest bond strength belonged to brackets bonded with CPP-ACP-containing adhesive after thermocycling. The reason for this increase could be alterations in the ACP structure in different thermal conditions. According to a study by Dorozhkin [41], amorphous agents like ACP tend to convert into stable crystallized structures (similar to hydroxyapatite) in a thermal range of 20–40°C, which could lead to reinforcement of the material’s structure [41]. Since thermal stresses are inevitable in the oral cavity during fixed orthodontic treatment, adding ACP to GIC would provide sufficient bond strength to resist forces applied to posterior teeth. It is necessary to emphasize that this was an in vitro study, and further clinical investigations are also required.

## CONCLUSION

Incorporating ACP into GIC resulted in a significant decrease in retentive bond strength before thermocycling. Also, thermocycling significantly decreased the retentive bond strength of specimens cemented with GIC. However, the retentive bond strength values significantly enhanced for specimens cemented with ACP-containing GIC after thermocycling.
